# Comparison of Adhesin Genes and Antimicrobial Susceptibilities between Uropathogenic and Intestinal Commensal *Escherichia coli* Strains

**DOI:** 10.1371/journal.pone.0061169

**Published:** 2013-04-09

**Authors:** Xiaohua Qin, Fupin Hu, Shi Wu, Xinyu Ye, Demei Zhu, Ying Zhang, Minggui Wang

**Affiliations:** 1 Huashan Hospital and Key Laboratory of Medical Molecular Virology of the Ministries of Education and Health, Shanghai Medical College, Fudan University, Shanghai, China; 2 Department of Molecular Microbiology and Immunology, Bloomberg School of Public Health, Johns Hopkins University, Baltimore, Maryland, United States of America; University of Malaya, Malaysia

## Abstract

The presence of adhesins is arguably an important determinant of pathogenicity for Uropathogenic *Escherichia coli* (UPEC). Antimicrobial susceptibilities were tested by agar dilution method, fifteen adhesin genes were detected by polymerase chain reaction, and multilocus sequence typing (MLST) was analyzed in 70 UPEC isolates and 41 commensal *E. coli* strains. Extended-spectrum β-lactamase (ESBL) was determined with confirmatory test. The prevalence of ESBL-producers in UPEC (53%, 37/70) was higher than the commensal intestinal isolates (7%, 3/41), and 97% (36/37) of the ESBL-producing UPEC harbored *bla*_CTX-M_ genes. *afa* was present in 36% (10/28) UPEC isolates from recurrent lower urinary tract infection (UTI), and none in the acute pyelonephritis, acute uncomplicated cystitis or commensal strains (*P*<0.0001). *papG* was detected in 28% (20/70) of UPEC isolates, while 5% (2/41) of the commensal strains were *papG* positive (*P* = 0.0025), and the prevalence of *papG* was significantly higher in acute pyelonephritis group (71%) than the other two UTI groups (*P*<0.0001). The prevalence of *flu*, *yqi, yadN* and *ygiL* was significantly higher in UPEC isolates than in the commensal strains. ESBL-producing UPEC showed a lower prevalence of adhesin genes compared with non-ESBL-producing strains. The MLST profiles were different between UPEC and commensal strains, with ST131 (19%, 13/70) and ST10 (20%, 8/41) being the most common MLSTs, respectively. This study demonstrated that several adhesin genes were more prevalent in UPEC isolates than in commensal *E. coli*, and *afa* may be associated with recurrent lower UTI whereas *papG* is more frequently associated with acute pyelonephritis.

## Introduction

Urinary tract infection (UTI) is one of the most common bacterial infections, and it is estimated that the overall lifetime prevalence of UTI in women is greater than 50% [1]. Further recurrent UTIs are reported in about 25% of women within 6 months of an acute UTI episode and pose a major health problem [2]. Uropathogenic *Escherichia coli* (UPEC) is the causative pathogen in 70–95% community-acquired UTI and over 50% nosocomial UTI [3]. Many virulence factors contribute to the pathogenesis of UPEC. The presence of adhesins is arguably the most important determinant of the pathogenicity for UPEC [4]. Adhesive organelles, including type 1, P, S/F1C, M, G and curli fimbriae, along with Afa/Dr adhesins, as well as temperature sensitive haemagglutinin (TSH), promote both bacterial attachment to and invasion of host tissues within the urinary tract [4]–[6].

*E. coli* expressing Dr/Afa adhesins may predispose the establishment of chronic or recurrent infections [7]. In previous studies, it was shown that UPEC entered the urinary epithelial cells through adhesins AfaD and AfaE, and therefore avoided the clearance of host immunosurveillance and antibiotic treatment. When the host cells shed, releasing a large amount of intracellular UPEC, a new round of infection could begin causing recurrence or relapse of UTI [8]. P fimbriae is encoded by the *pap* gene cluster, including *papG* encoding the tip adhesin PapG [9]. The three most studied PapG molecular variants, which are shown to bind distinct isoreceptors are PapG I, II and III [10]. Clinically the class II *papG* allele is primarily associated with human pyelonephritis, and class III *papG* allele with genitourinary infections in dogs and cats yet some cases of human cystitis [11]. In addition to P fimbriae, type 1 and S/F1C fimbriae, curli and M and G fimbriae which are encoded by the *fim and sfa/foc, csg, bma* and *gaf* gene clusters, respectively, may also be related to the adherence of UPEC [4], [6], [12]. More recently, it is discovered that adhesin Ag43, encoded by *flu* and abundant on the outer membrane of *E. coli*, enables bacterial cells to adhere to each other and form biofilm over the host urothelial cells, leading to poor clearance of the pathogens [13]. Other novel genes like *yqi, yadN and ygiL* were also found to be prevalent among UPEC strains and played an important role in colonization and pathogenesis [6], [14].

Previous studies have shown that the prevalence of adhesin genes is different between UPEC and fecal commensal strains of *E. coli* [15]. However, few epidemiologic data about adhesin genes of UPEC are available in China. The aim of the present study was to compare the prevalence of the adhesin genes and antimicrobial susceptibilities between UPEC and intestinal colonizing strains of *E. coli*, and adhesin genes between strains isolated from different types of UTIs and their association with a particular type of UTIs.

## Materials and Methods

### *E. coli* isolates

A total of 70 clinical isolates of UPEC were collected from clean midstream urine specimens of inpatients (63 female and 7 male) with urinary tract infections at Huashan Hospital, Shanghai from January 2008 to December 2010. The exclusion criteria were: 1) patients with indwelling catheter; 2) patients with urinary calculus or structural abnormalities. Isolation was performed according to standard laboratory protocols and UPEC were isolated from urine with >10^5^ colony-forming units/ml.

Among the 70 UPEC isolates, 28 were from patients with recurrent lower UTIs. The recurrent lower UTIs were defined as at least two episodes of UTI within 6 month. Each time the patients had: a) typical clinical symptoms as urinary urgency, pain, and frequent voiding, b) pyuria, c) without fever, chill or other systematic symptoms. All of the 28 patients in recurrent lower UTI group had recurrences caused by UPEC. MLST typing of the UPEC strains from 11 randomly selected patients showed that the strains isolated from different episodes of each patient had the same MLST type.

Seventeen strains were isolated from acute pyelonephritis patients by the following criteria: a) the temperature >38.5°C, b) positive percussion pain on kidney area, c) an elevated leukocyte count (>11×10^9^/L) and elevated percentage of neutrophils (>70%) in the blood routine test. The remaining 25 strains were from patients with acute uncomplicated cystitis.

The intestinal commensal *E. coli* isolates (n = 41) were collected from anal swab specimens of healthy volunteers. The inclusion criteria emphasized: 1) without diarrhea or abdominal pain, 2) without antibiotic administration within the three months before taking the specimen. After isolation, all isolates were kept frozen at −70°C in 20% glycerol.

### Antimicrobial susceptibility testing

The minimum inhibitory concentrations (MICs) of imipenem, cefepime, ceftazidime, cefotaxime, cefuroxime, cefazolin, piperacillin/tazobactam, piperacillin, gentamicin, amikacin, ciprofloxacin, and nitrofurantoin for both UPEC and commensal isolates were determined by agar dilution according to CLSI standards [16]. The antimicrobial concentrations were from 0.06 to 128 µg/ml. *E. coli* ATCC 25922 was used as a quality control strain.

### Detection of ESBL-producing bacterial strains and ESBL genes

Extended-spectrum β-lactamase (ESBL) was determined with the previously recommended CLSI disk diffusion ESBL screening and confirmatory test [17]. CTX-M- specific genes in ESBL-producing strains were identified by polymerase chain reaction (PCR) with the primers reported previously [18].

### Adhesin gene detection

The presence of genes *afa, draE, daaE, papG I, papG II, papG III, flu, fimH, sfaD, focG, bmaE, gafD, csgA, tsh, yqi*, *yadN* and *ygiL* was investigated by PCR amplification using primers (Sangon, Shanghai, China) as described previously [12], [14], [19]–[22]. The sequence amplified by *afa* primers, located between *afaB* and *afaC*, was conservatively shared by many subtypes of Dr/Afa adhesins [19]. All the *sfa* and *foc* PCR fragments were confirmed by sequencing as *sfa* and *foc* are highly homologous. The positive isolates identified by sequencing of PCR products in the pre-test were used as positive control in the following screening.

### Analysis of UPEC by multilocus sequence typing (MLST)

All of the 70 clinical UPEC and 41 intestinal commensal isolates were analyzed by MLST. The seven housekeeping genes (*adk*, *fumC*, *icd*, *purA*, *gyrB*, *recA* and *mdh*) for typing [23], as well as the primer sets and PCR conditions were according to the MLST website for *E. coli* (mlst.ucc.ie/mlst/dbs/Ecoli/documents/primersColi_html). The based upon related sequence types (BURST) clustering algorithm (eburst.mlst.net) was used to analyze the allelic profiles and define clonal complexes (CCs). CCs were identified according to the number of single-locus variants (SLVs) and double-locus variants (DLVs) shared between isolates, where only STs that shared six or more loci were assigned to a defined CCs.

### Statistical analysis

The comparison of prevalence rates was performed using Pearson Chi-square test or Fisher's exact test (two-tailed) with the software SPSS 16.0, and the level of significance was set at *P*<0.05.

## Results

### Antimicrobial susceptibility and ESBL differences of the two groups of *E. coli* strains

UPEC isolates demonstrated remarkably lower susceptibility rates to piperacillin (14%), cefazolin (21%), cefuroxime (39%), cefotaxime (43%), ceftazidime (71%), cefepime (79%), gentamicin (43%), amikacin (90%) and ciprofloxacin (23%) than intestinal commensal isolates (*P*<0.05). However, 91%–100% of *E. coli* isolates in the two groups were susceptible to piperacillin/tazobactam, imipenem and nitrofurantoin and there was no significant difference between the two groups (*P*>0.05, Table 1).

**Table 1:** Comparison of the antimicrobials susceptibility between UPEC and commensal *E. coli* strains.

Antimicrobial agents	UPEC isolates (n = 70)				Commensal *E. coli* strains (n = 41)				*P* value
	MIC range (µg/ml)	MIC_50_	MIC_90_	S%	MIC range (µg/ml)	MIC_50_	MIC_90_	S%	
Piperacillin	1–256	128	256	14	0.5–256	2	64	73	<0.0001
Piperacillin/tazobactam	0.25–256	2	4	91	0.5–16	1	2	100	0.0539
Cefazolin	0.06–256	128	256	21	0.5–256	1	64	83	<0.0001
Cefuroxime	2–256	256	256	39	2–256	2	256	85	<0.0001
Cefotaxime	0.06–256	8	256	43	0.06–128	0.06	8	85	<0.0001
Ceftazidime	0.06–256	0.5	32	71	0.06–32	0.125	0.25	98	0.0007
Cefepime	0.06–256	1	16	79	0.06–8	0.06	1	100	0.0014
Imipenem	0.06–0.25	0.06	0.125	100	0.06–125	0.06	0.06	100	-
Gentamicin	0.25–256	64	128	43	0.5–256	1	64	85	<0.0001
Amikacin	1–256	2	8	90	1–4	2	2	100	0.0364
Ciprofloxacin	0.06–256	32	128	31	0.06–16	0.06	1	90	<0.0001
Nitrofurantoin	4–128	8	16	99	4–32	8	16	100	0.4420

Thirty-seven (53%) of 70 UPEC isolates, while 3 (7%) out of 41 intestinal commensal isolates were ESBL-producing (*P*<0.001). *bla*_CTX-M_ genes were present in 97% (36/37) ESBL-producing isolates, and of these, 58% (21/36) carried *bla*_CTX-M-1_-group ESBL genes while 42% (15/36) belonged to *bla*_CTX-M-9_-group. ESBL genotype could not be determined in one strain. The CTX-M-2, CTX-M-8 and CTX-M-25/26 groups were not found.

### Prevalence of adhesin genes

#### (1) Afa/Dr adhesin family member genes

*afa* was present only in 10 UPEC isolates that belonged to the recurrent lower UTIs group (10/28, 36%). None of the isolates from the acute pyelonephritis group or the acute uncomplicated cystitis group carried *afa*. No *afa* was found in 41 intestinal commensal *E. coli* isolates. Neither *draE* nor *daaE* was detected among all the strains we screened.

#### (2) Type P fimbriae related gene *papG*

*papG* was positive in 28% (20/70) of the UPEC isolates including 16 *papG II* and 4 *papG III*, and 5% (2/41) of the intestinal commensal isolates carried *papG II* (*P* = 0.0025). The prevalence of *papG* in acute pyelonephritis (71.4%, 5/7) was significantly higher than that of recurrent lower UTI group (14.3%, 3/21, *P* = 0.0004) and that of acute uncomplicated cystitis group (15.8%, 3/19, *P* = 0.0001). No *papG I* was detected in any of all the tested strains.

#### (3) Biofilm related adhesin gene *flu* and novel genes *yqi, yadN* and *ygiL*

A majority (77%, 54/70) of the UPEC isolates and 41% (17/41) of the intestinal commensal isolates were found to be *flu* positive (*P* = 0.0002). The prevalence of novel genes *yqi, yadN* and *ygiL* was higher in UPEC isolates than that of the commensal strains (27% *vs* 7%, 54% *vs* 32%, and 44% *vs* 17%, respectively, *P*<0.05). The prevalence differences of these four genes of *flu*, *yqi*, *yadN* and *ygiL* between the three UTI groups were not significant.

#### (4) Type 1 fimbrial gene *fimH* and curli fiber gene *csgA*

*fimH* was found positive in 86% (60/70) UPEC isolates and 73% (30/41) commensal isolates (*P* = 0.1034). The positive rates of *fimH* in the three UTI groups were 86%, 82% and 88%, respectively. 30% (21/70) UPEC isolates and 34% (14/41) commensal ones carried *csgA* (*P* = 0.6500).

#### (5) Other adhesin genes

*sfaD* was present in 5 isolates and *focG* in 2 isolates among the UPEC group. One of the *sfaD* carrying isolates was also detected *focG* positive. None of the isolates in the intestinal commensal group were *sfaD*/*focG* positive. No more than 5 isolates in UPEC or commensal groups produced amplicons with the *bmaE*, *gafD* or *tsh* primers. (Table 2)

**Table 2:** Comparison of the prevalence of adhesin genes among different groups of *E. coli* strains.

Adhesin genes^a^	Recurrent lower UTI, n = 28 (%)	Acute pyelonephritis, n = 17 (%)	Acute uncomplicated cystitis, n = 25 (%)	Total UPEC, n = 70 (%)	Commensal isolates, n = 41(%)	*P* value (Total UPEC *vs* Commensal)
*afa*	36^b^	0	0	14	0	<0.0001^c^
*papG*	18	71^d^	12	28	5	0.0025
*flu*	89	65	72	77	41	0.0002
*fimH*	86	82	88	86	73	0.1034
*sfaD*	4	12	8	7	0	0.0799
*focG*	0	12	0	3	0	0.2747
*bmaE*	4	6	4	4	5	0.8845
*gafD*	4	0	0	1	5	0.2794
*csgA*	21	24	44	30	34	0.6500
*tsh*	0	18	8	7	5	0.6357
*yqi*	21	35	28	27	7	0.0114
*yadN*	50	76	44	54	32	0.0212
*ygiL*	46	43	40	44	17	0.0035

a , *draE* and *daaE* genes were not detected in any of the isolates.

b , Compared to the other two UTI groups, *P*<0.0001;

c . Recurrent lower UTI group compared to commensal isolates;

d , Compared to the other two UTI groups, *P*<0.0001.

### Comparison of the prevalence of fimbrial genes between ESBL-producing and non-ESBL-producing UPEC

The prevalence of *fimH* (97%, 32/33) in the non-ESBL-producing UPEC was significantly higher than that in the ESBL-producing UPEC (76%, 28/37, *P* = 0.0110). All the six strains carrying *sfaD/focG* were from the non-ESBL-producing UPEC group. The prevalence of the other fimbrial genes in the non-ESBL-producing UPEC group seemed a little higher than the ESBL-producing UPEC strains, however, there were no statistically significant differences (*P*>0.05, Table 3)

**Table 3:** Comparison of the prevalence of fimbrial genes between ESBL-producing and non-ESBL-producing UPEC strains.

Fimbrial genes	ESBL-producing UPEC n = 37 (%)	Non-ESBL-producing UPEC n = 33 (%)	*P* value
*afa*	14	15	0.8450
*papG*	22	36	0.1729
*flu*	73	82	0.3790
*fimH*	76	97	0.0110
*sfaD/focG*	0	18	0.008
*bmaE*	3	6	0.5990
*gefD*	0	3	0.4710
*csgA*	22	39	0.1053
*tsh*	5	9	0.6610
*yqi*	22	33	0.2714
*yadN*	49	61	0.3161
*yqiL*	43	45	0.8525

### MLST analysis of UPEC and commensal isolates

**Figure 1:**
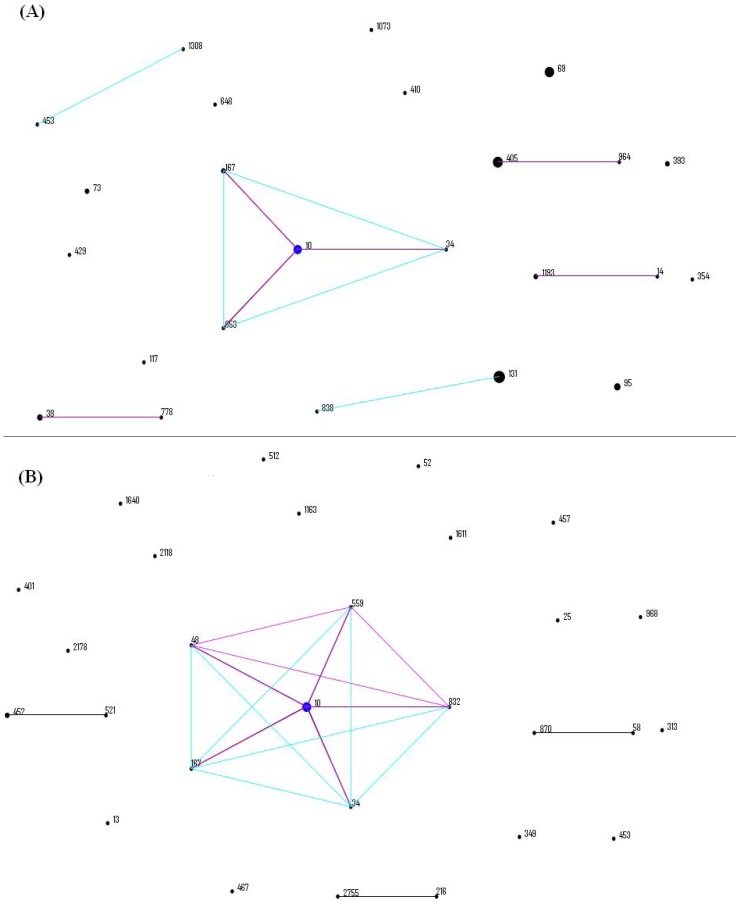
eBURST diagrams of UPECand intestinal commensal isolates showing related STs and individual STs. (A) referred to UPEC isolates and (B) referred to intestinal commensal isolates. Each ST is represented by a circle, the size of which correlates to the frequency of the ST. Predicted founders are positioned centrally and shown in blue, and single-locus variants and double-locus variants are shown in pink and blue, respectively.

The 70 UPEC isolates analyzed were assigned to 26 distinct STs. The most common STs were ST131 (n = 13, 19%), followed by ST69 (9, 13%), ST405 (8, 11%), ST10 (7, 10%), ST393 (4, 6%), ST95 (4, 6%) and ST38 (3, 4%). The seven most common STs accounted for 69% (48/70) of the isolates, demonstrating the diversity of lineages. eBURST analysis revealed that 4 CCs (CC10, CC405, CC14 and CC38) encompassing 10 STs represented 25 (36%) UPEC isolates and the remaining 45 isolates included in the other 16 STs appearing as singletons (Figure 1A).

The 41 intestinal commensal isolates analyzed by MLST were grouped into 32 different STs. 28 were already included in the *E. coli* website database and 4 were new STs. ST10 (n = 8, 20%) was the most common group. A new ST had 2 strains, while the other 29 STs contained a single strain each. As analyzed by eBURST, ST10, ST34, ST167, ST48, ST559 and ST832 belonged to CC10 (Figure 1B).

## Discussion

Most of the intestinal commensal isolates tested in this study were susceptible to all the tested antimicrobial agents, while the UPEC isolates were characterized by a varying degree of susceptibilities to different antimicrobials. Of 70 UPEC strains, 37 (53%) produced extended-spectrum β-lactamase (ESBL). In a previous study, 2.9% of *E. coli* isolates recovered by outpatient urine cultures in 2008 harbored ESBL in USA [24], which is much higher than previously reported but far lower than the rate in our study. The annual percentage of ESBL-producing UPEC with *bla*_CTX-M_ genes changed from 35% in 2003 to 64% in 2008 with the average rate of 56% in that study [24]. However, in this study, *bla*_CTX-M_ ESBL genes were detected in 36 (97%) of 37 ESBL positive strains, which is much higher than in the USA.

All 10 UPEC isolates harboring the *afa* belonged to recurrent lower UTI group, none was *afa* positive in isolates from other UTIs or intestinal carriage, indicating that UPEC carrying *afa* might correlate with the recurrence of UTI episodes. Blanco and colleagues screened UPEC for *afa* and found no *afa* positive strain in acute pyelonephritis isolates, however, *afa* was detected in 5 of the 116 isolates (4.3%) from cystitis patients and 1 of the 42 (2.4%) isolates from asymptomatic bacteriuria [19]. Previous studies showed that Afa/Dr fimbrial adhesins contributed to the ability of UPEC isolates to colonize and persist long term within the urinary tract and therefore more likely to cause the recurrence of UTI episodes [7]–[8].

The isoreceptors of adhesin PapG primarily are scattered within the kidney, including glomerulus, proximal tubulus, distal tubulus, and collecting duct. Therefore sitting on the top of type P fimbriae, adhesin PapG is thought to be associated with bacterial adhesion to the kidney [9]. The prevalence of *papG* in acute pyelonephritis isolates was significantly higher (71%) than that in recurrent lower UTIs (18%) and acute uncomplicated cystitis (12%) in the present study, illustrating high correlation between the *papG* and pyelonephritis. Our findings are consistent with some previous studies. Johnson et al found 69% (118/170) acute pyonephritis strains and 25% (21/83) of cystitis strains were *papG* positive [15].

Encoded by *flu*, Ag43 is a self-recognizing adhesin that is associated with cell aggregation and biofilm formation of UPEC [13]. We found a high prevalence (77%) of *flu* in UPEC isolates, while *flu* was detected in 41% of the intestinal commensal isolates. The finding is consistent with the results of Ulett and colleagues. They reported 83% (30/36) of the UPEC isolates and 56% (35/62) of the commensal strains carried *flu* [22]. However, there was no statistically significant difference in *flu* gene prevalence between recurrent lower UTI and non-recurrent lower UTI in the present study.

It was reported that the adhesin encoding gene *yqi* was prevalent among UPEC by more than 50% while absent in all the intestinal pathogenic *E. coli* [6]. In this study, *yqi* was detected in 7% of commensal isolates, but the prevalence was significantly lower than that in UPEC strains (27%, *P* = 0.0114).

*yadN* and *ygiL* were found to be more prevalent in UPEC (54% and 44%, respectively) than the intestinal commensal strains (32% and 17%) in the present study (*P*<0.05), which coincides with Mobley's work. It was reported that fimbriae related structure Yad and Ygi could enhance the virulence-related phenotypes, including biofilm formation and adherence to immortalized human epithelial cells [14]. In addition, we did not detect any significant prevalence difference of *yadN* or *ygiL* between UPEC strains causing recurrent lower UTI, acute pyelonephritis and uncomplicated cystitis.

The results showed that 86% of UPEC isolates and 73% of commensal isolates carried *fimH*, encoding the adhesin FimH as the tip fimbria for type 1 fimbriae. Wang et al detected 91% (72/79) *fimH* positive in UPEC isolates [25]. On the other hand, Schlager found that 81% (74/91) strains isolated from fecal specimen of healthy girls carried *fimH* [26]. A high prevalence of *fimH* was found in both UPEC and commensal isolates. However, the transcription and expression level of *fimH* might be different between UPEC and commensal strains, which made type 1 fimbriae an important virulence factor for UPEC [27].

In the present study, we found that ESBL-producing UPEC isolates showed a lower prevalence of fimbrial genes, *fimH* and *sfaD/focG*, compared with non-ESBL-producing ones. The results coincided with the previous findings that fimbrial genes were of reduced prevalence among UPEC resistant to extended-spectrum cephalosporins. A possible explanation is that: virulence traits like the fimbrial genes, as well as resistance genes, can be carried on conjugative plasmids; therefore, the incompatible resistance-encoding plasmids are outcompeting fimbrial factor encoding plasmids [28]. It is reported that the acquisition of antibiotic resistance may cause alterations in phenotypic and physiological characteristics, which are referred to as “biological fitness cost”. The biological fitness cost on antibiotics resistance generally results in reduced growth rates. More dramatic phenotypic changes, including poor fimbrial expression, were reported in an ampicillin-resistant mutant *Acinetobacter* sp. strain DR1A comparing with the wild type strain DR1 [29]. Therefore, the decreased fimbrial genes and adherence capability in ESBL-producing UPEC might also be a fitness trade-off for the ESBL to survive antibiotics exposure. The exact explanation for the lower incidence of fimbrial genes among resistant UPEC isolates needs additional study.

In this study, 69% of the UPEC isolates were included in the seven STs (ST131, ST69, ST405, ST10, ST393, ST95 and ST38) and ST131 was the most predominant (19%). Gibreel revealed a consistent profile of STs over a 3-year period with UPEC isolates (n = 300) in the Northwest region of England primarily from 8 lineages: ST73 (16.6%), ST131 (12.3%), ST69 (9%), ST95 (6.3%), ST10 (4.3%), ST127 (3.6%), ST14 (2.7%) and ST405 (1.7%) [30]. Most of these STs (ST131, ST69, ST95, ST10 and ST405) were also prevalent in our study. The 41 commensal isolates had a richer ST diversity (32 STs) than the 70 UPEC counterparts (26 STs). A ST10 *E. coli* strain was common in UPEC (10%) as well as in intestinal commensal isolates (20%). One isolate from each group was ST453. The remaining 24 STs of the UPEC isolates were different from the other 30 STs of the commensal strains.

This study indicated that *afa* of Afa/Dr adhesin family might be associated with lower UTI recurrence, while *papG* might be correlated with acute pyelonephritis. However, we only focused on detecting the prevalence of adhesin genes by PCR, instead of the mechanism of the related adhesins of UPEC. Therefore, we could not prove the correlation between the adhesins and the pathogenesis of the UPEC. Moreover, because of the “cross-talk” among the regulators of different adhesin systems of UPEC and their functional redundancy, when a certain type of adhesin is lost, it may be quite difficult to study the relationship between the UTI recurrence and a specific kind of adhesin. Studies on other factors and mechanisms involved in pathogenesis and persistence of UPEC are required.
